# Supplementary material for: Nest architecture and colony composition in two populations of Ectatomma ruidum sp. 2 (E. ruidum species complex) in southwestern Colombia
Source: PLoS One. 2022 Feb 2;17(2):e0263382. doi: 10.1371/journal.pone.0263382 (PMC8809609; doi:10.1371/journal.pone.0263382)
Supplement: S1 Table — Nests 1–24 were extracted using the paraffin wax technique. Q: queens; G: gynes; M: males; W: workers; P: pupae; L: larvae. The number of chambers was considered only for the nests collected at ‘Cali’ (103 nests). Nests 36, 44, 48, 52, 60, 72, 76, 81, 84, 88, 96, 99, 104, 106, and 114, containing fewer than 10 workers and no sexual adults and brood, were considered abandoned and were not considered for subsequent analysis. They are not reported in this table. (PDF) [file pone.0263382.s001.pdf]

Table S1. Composition of the population of the *E. ruidum* sp. 2 nests extracted at the University Campus of the Universidad del Valle ('Cali') and at Vereda El Rosal, Caldono ('Cauca'). Nests 1-24 were extracted using the paraffin wax technique. Q: queens; G: gynes; M: males; W: workers; P: pupae; L: larvae. The number of chambers was considered only for the nests collected at 'Cali' (103 nests). Nests 36, 44, 48, 52, 60, 72, 76, 81, 84, 88, 96, 99, 104, 106, and 114, containing fewer than 10 workers and no sexual adults and brood, were considered abandoned and were not considered for subsequent analysis. They are not reported in this table.

Nest	Q	G	M	W	P	L	Nest depth (cm)	Number of chambers	Entrance diameter (mm)	Site
1	0	0	3	42	0	0	32	7	5.0	Cali
2	0	1	5	77	3	1	25	3	3.5	Cali
3	0	1	2	78	24	7	28	7	4.0	Cali
4	0	4	1	51	1	0	31	8	3.0	Cali
5	0	12	3	72	12	6	36	9	3.0	Cali
6	0	0	9	72	17	9	27	6	5.0	Cali
7	1	0	0	152	0	0	34	7	3.0	Cali
8	0	2	3	42	5	3	31	6	4.0	Cali
9	0	0	1	58	10	8	25	6	4.5	Cali
10	0	1	4	97	18	7	31	7	3.5	Cali
11	0	2	1	74	7	4	35	6	3.0	Cali
12	0	1	6	62	18	15	36	8	5.0	Cali
13	0	0	5	71	6	5	32	8	4.5	Cali
14	0	1	2	71	10	8	23	5	4.5	Cali
15	0	1	3	36	7	3	28	6	4.5	Cali
16	0	1	7	75	29	20	24	6	3.5	Cali
17	1	1	0	125	25	17	29	8	5.0	Cali
18	0	0	1	22	1	0	22	4	4.0	Cali
19	0	3	0	71	17	15	28	5	5.0	Cali
20	0	0	2	26	7	0	32	6	5.0	Cali
21	0	0	6	14	0	0	25	5	3.5	Cali
22	0	0	7	47	13	9	29	5	5.0	Cali
23	0	1	1	27	3	0	30	7	5.0	Cali
24	0	0	3	10	0	0	26	3	4.0	Cali
25	0	2	6	73	31	23	28	3	-	Cali
26	1	0	0	148	21	80	34	8	-	Cali
27	0	3	2	101	39	43	26	5	-	Cali
28	0	0	2	94	35	32	28	3	-	Cali
29	1	1	0	120	24	76	31	7	-	Cali
30	0	2	0	84	8	15	28	3	-	Cali
31	0	1	3	59	17	25	27	4	-	Cali

32	0	2	2	67	3	41	31	8	-	Cali
33	0	1	6	63	13	27	27	3	-	Cali
34	0	2	5	75	15	25	32	5	-	Cali
35	0	0	6	70	36	21	22	3	-	Cali
37	1	0	0	64	8	36	19	5	-	Cali
38	0	0	2	40	2	15	23	6	-	Cali
39	0	0	3	127	18	53	28	6	-	Cali
40	0	0	1	57	14	25	19	5	-	Cali
41	0	2	0	82	8	32	32	5	-	Cali
42	0	1	2	65	10	27	21	4	-	Cali
43	0	0	0	48	13	19	23	4	-	Cali
45	0	0	1	27	0	9	24	3	-	Cali
46	0	3	0	40	4	10	32	5	-	Cali
47	1	0	2	176	7	88	38	7	-	Cali
49	0	0	3	78	20	36	28	6	-	Cali
50	0	0	6	51	28	40	32	7	-	Cali
51	0	0	1	39	6	16	32	4	-	Cali
53	0	0	3	54	18	25	37	6	-	Cali
54	0	0	0	74	2	26	35	6	-	Cali
55	1	0	0	190	2	92	34	8	-	Cali
56	0	0	0	38	6	18	25	5	-	Cali
57	0	0	5	72	33	36	31	7	-	Cali
58	0	2	3	55	6	37	32	6	-	Cali
59	0	1	0	44	2	29	33	8	-	Cali
61	0	0	0	30	11	63	28	6	-	Cali
62	0	0	0	40	4	16	24	5	-	Cali
63	0	0	0	52	13	23	19	4	-	Cali
64	0	1	0	55	3	38	21	5	-	Cali
65	0	0	0	56	13	25	31	7	-	Cali
66	0	0	0	42	4	15	30	5	-	Cali
67	0	0	1	51	2	11	38	9	-	Cali
68	0	1	1	74	14	41	23	4	-	Cali
69	1	1	0	150	22	68	30	7	-	Cali
70	0	0	0	21	0	0	23	4	-	Cali
71	0	0	1	53	9	16	26	5	-	Cali
73	0	1	0	52	2	22	24	4	-	Cali
74	0	1	1	51	0	13	30	5	-	Cali
75	0	1	2	67	10	63	27	8	-	Cali
77	0	0	4	67	11	30	25	4	-	Cali
78	0	4	0	101	4	32	29	6	-	Cali
79	0	3	0	45	4	11	23	4	-	Cali

80	0	1	0	44	0	0	28	5	-	Cali
82	0	0	2	84	11	41	28	6	-	Cali
83	0	0	0	47	9	53	23	5	-	Cali
85	0	1	0	92	15	37	26	6	-	Cali
86	0	0	0	46	4	6	20	2	-	Cali
87	0	0	0	56	10	33	28	6	-	Cali
89	0	1	0	52	4	16	35	8	-	Cali
90	0	0	0	63	7	24	31	7	-	Cali
91	0	0	2	26	2	0	25	4	-	Cali
92	1	1	0	127	12	45	23	4	-	Cali
93	0	0	0	60	15	20	37	9	-	Cali
94	0	0	1	53	21	31	29	6	-	Cali
95	0	0	0	57	13	32	24	6	-	Cali
97	0	0	0	39	1	15	28	3	-	Cali
98	1	1	0	52	7	23	24	6	-	Cali
100	0	0	0	16	0	0	26	5	-	Cali
101	0	0	4	68	16	48	35	6	-	Cali
102	0	2	1	98	15	5	33	7	-	Cali
103	0	2	0	54	31	37	28	7	-	Cali
105	0	0	3	61	13	28	27	7	-	Cali
107	0	1	1	59	12	32	28	7	-	Cali
108	0	2	2	54	36	48	32	6	-	Cali
109	0	0	0	26	1	48	35	7	-	Cali
110	1	0	0	132	2	73	32	8	-	Cali
111	0	0	0	23	0	10	31	7	-	Cali
112	0	3	1	53	23	51	38	7	-	Cali
113	0	1	4	77	36	54	32	7	-	Cali
115	0	1	6	26	1	48	31	7	-	Cali
116	0	1	6	52	3	36	32	6	-	Cali
117	0	3	5	72	51	42	30	8	-	Cali
118	1	0	0	115	5	0	38	6	-	Cali
119	0	0	0	40	10	52	28	-	-	Cauca
120	1	0	13	179	110	147	39	-	-	Cauca
121	1	0	4	211	75	120	80	-	-	Cauca
122	1	0	0	202	89	91	44	-	-	Cauca
123	0	0	0	21	12	30	21	-	-	Cauca
124	1	0	0	127	10	103	26	-	-	Cauca
125	0	0	0	123	0	50	24	-	-	Cauca
126	0	0	0	33	18	17	29	-	-	Cauca
127	0	0	0	56	9	60	37	-	-	Cauca
128	1	0	0	52	42	40	28	-	-	Cauca

129	0	1	3	35	26	20	28	-	-	Cauca
130	1	0	0	158	92	130	42	-	-	Cauca
131	0	0	0	97	52	42	47	-	-	Cauca
132	1	0	0	42	33	49	30	-	-	Cauca
133	1	0	0	134	97	175	46	-	-	Cauca
134	0	0	0	119	160	52	27	-	-	Cauca
135	0	0	0	65	59	19	43	-	-	Cauca
136	1	0	0	114	15	154	49	-	-	Cauca
137	1	0	0	116	60	59	25	-	-	Cauca
138	0	0	0	57	11	92	23	-	-	Cauca
139	0	0	2	70	5	35	30	-	-	Cauca
140	0	0	0	120	18	53	37	-	-	Cauca
141	1	0	0	127	56	82	35	-	-	Cauca
142	1	0	3	120	56	100	33	-	-	Cauca
143	1	0	7	116	70	47	27	-	-	Cauca
144	1	0	0	125	75	86	46	-	-	Cauca
145	0	0	0	49	26	28	26	-	-	Cauca
146	1	0	0	190	181	203	65	-	-	Cauca
147	0	2	13	137	37	62	48	-	-	Cauca
148	0	0	1	85	45	45	30	-	-	Cauca
149	1	1	5	137	125	92	32	-	-	Cauca
150	0	0	1	130	77	144	57	-	-	Cauca
151	1	0	0	144	120	86	42	-	-	Cauca
152	0	0	0	45	4	34	22	-	-	Cauca
153	0	0	0	74	17	28	36	-	-	Cauca
154	0	0	0	98	25	50	36	-	-	Cauca
155	0	0	0	59	20	64	35	-	-	Cauca
156	0	0	0	46	0	6	24	-	-	Cauca
157	0	0	0	43	9	62	26	-	-	Cauca
158	1	0	0	212	88	95	65	-	-	Cauca
159	0	0	0	33	27	69	23	-	-	Cauca
160	0	0	0	39	26	39	27	-	-	Cauca
161	0	0	0	85	67	43	31	-	-	Cauca
162	0	0	3	30	57	43	25	-	-	Cauca
163	1	0	0	166	194	201	41	-	-	Cauca
164	1	0	0	177	150	151	40	-	-	Cauca
165	1	0	0	59	37	55	24	-	-	Cauca
166	0	0	0	69	119	81	26	-	-	Cauca
167	0	0	0	92	81	51	29	-	-	Cauca
