# Supplementary material for: Nest architecture and colony composition in two populations of Ectatomma ruidum sp. 2 (E. ruidum species complex) in southwestern Colombia
Source: PLoS One. 2022 Feb 2;17(2):e0263382. doi: 10.1371/journal.pone.0263382 (PMC8809609; doi:10.1371/journal.pone.0263382)
Supplement: S2 Table — Q: queens; G: gynes; M: males; W: workers; P: pupae; L: larvae. (PDF) [file pone.0263382.s002.pdf]

Table S2. Chamber dimensions (in cm), volume (in cm^3), and population within each chamber for the 24 *E. ruidum* sp. 2 nests extracted using the paraffin wax technique at 'Cali'. Q: queens; G: gynes; M: males; W: workers; P: pupae; L: larvae.

Nest	Size	Chambers								
		I	II	III	IV	V	VI	VII	VIII	IX
1	Width	5.2	6.0	3.6	4.0	3.1	3.5	3.6	-	-
	Length	9.3	11.1	5.4	6.3	4.4	5.0	4.8	-	-
	Height	1.9	2.2	1.8	2.0	1.8	2.0	1.7	-	-
	Depth	6.9	10.2	12.8	16	20.8	26	31.7	-	-
	Volume	24.1	38.4	9.2	13.2	6.4	9.2	7.7	-	-
	Q	-	-	-	-	-	-	-	-	-
	G	-	-	-	-	-	-	-	-	-
	M	-	-	2	-	-	1	-	-	-
	W	2	-	11	-	2	26	1	-	-
	P	-	-	-	-	-	-	-	-	-
	L	-	-	-	-	-	-	-	-	-
2	Width	7.0	6.8	4.5	-	-	-	-	-	-
	Length	10.8	12.0	6.4	-	-	-	-	-	-
	Height	2.3	2.0	1.8	-	-	-	-	-	-
	Depth	8.3	13	22.8	-	-	-	-	-	-
	Volume	45.5	42.7	13.6	-	-	-	-	-	-
	Q	-	-	-	-	-	-	-	-	-
	G	-	-	1	-	-	-	-	-	-
	M	1	4	-	-	-	-	-	-	-
	W	5	17	55	-	-	-	-	-	-
	P	3	-	-	-	-	-	-	-	-
	L	1	-		-	-	-	-	-	-
3	Width	5.6	5.0	4.1	3.2	3.4	2.4	2.0	-	-
	Length	10.5	10.2	7.6	4.1	4.0	3.3	3.0	-	-
	Height	2.3	2.0	1.8	1.8	2.0	1.5	1.5	-	-
	Depth	5.3	7	10.8	15.8	19	22.5	28.5	-	-
	Volume	35.4	26.7	14.7	6.2	7.1	3.1	2.4	-	-
	Q	-	-	-	-	-	-	-	-	-
	G	-	-	-	-	-	1	-	-	-
	M	2	-	-	-	-	-	-	-	-
	W	17	6	-	24	5	17	9	-	-
	P	18	6	-	-	-	-	-	-	-
	L	7	-	-	-	-	-	-	-	-
4	Width	4.6	4.0	3.8	4.1	3.5	3.0	2.8	2.0	-
	Length	8.2	6.3	5.0	6.9	5.0	3.8	3.5	2.0	-
	Height	1.8	2.0	2.0	1.8	1.9	2.0	1.9	1.8	-

	Depth	4.8	7	9	14.8	19.9	26	28.9	31.8	-
	Volume	17.8	13.2	10.0	13.3	8.7	6.0	4.9	1.9	-
	Q	-	-	-	-	-	-	-	-	-
	G	-	-	2	-	1	-	-	1	-
	M	1	-	-	-	-	-	-	-	-
	W	5	2	22	-	9	2	-	11	-
	P	-	1	-	-	-	-	-	-	-
	L	-	-	-	-	-	-	-	-	-
	Width	4.5	5.6	6.3	4.1	6.0	3.6	3.0	3.2	1.8
5	Length	7.7	9.4	11.0	6.2	11.5	5.3	5.2	4.1	2.6
	Height	2.2	2.0	2.3	2.1	2.2	2.0	1.8	1.8	1.8
	Depth	6.2	8	12.3	15.1	18.2	22	24.8	28.8	33.8
	Volume	20.0	27.6	41.7	14.0	39.7	10.0	7.4	6.2	2.2
	Q	-	-	-	-	-	-	-	-	-
	G	-	1	2	-	1	-	4	-	4
	M	-	-	3	-	-	-	-	-	-
	W	-	7	25	5	17	-	13	-	5
	P	-	-	2	10	-	-	-	-	-
	L	-	-	-	6	-	-	-	-	-
	Width	5.6	5.1	4.6	3.8	2.5	2.0	-	-	-
6	Length	8.3	7.4	6.3	5.4	3.5	2.2	-	-	-
	Height	2.3	2.1	2.1	2.0	1.6	1.5	-	-	-
	Depth	5.3	7.1	10.1	17	22.6	26.5	-	-	-
	Volume	28.0	20.8	15.9	10.7	3.7	1.7	-	-	-
	Q	-	-	-	-	-	-	-	-	-
	G	-	-	-	-	-	-	-	-	-
	M	-	3	5	1	-	-	-	-	-
	W	-	10	27	30	3	2	-	-	-
	P	-	-	17	-	-	-	-	-	-
	L	-	-	9	-	-	-	-	-	-
	Width	5.3	4.1	5.5	4.4	3.1	3.3	2.7	-	-
7	Length	7.4	5.6	10.2	8.5	4.1	4.8	3.8	-	-
	Height	2.4	2.1	2.2	2.0	1.7	1.8	1.9	-	-
	Depth	8.4	11.1	15.2	21	24.7	27.8	29.9	-	-
	Volume	24.6	12.6	32.3	19.6	5.7	7.5	5.1	-	-
	Q	1	-	-	-	-	-	-	-	-
	G	-	-	-	-	-	-	-	-	-
	M	-	-	-	-	-	-	-	-	-
	W	55	29	58	6	1	2	1	-	-
	P	-	-	-	-	-	-	-	-	-
	L	-	-	-	-	-	-	-	-	-
8	Width	4.6	4.3	1.7	1.4	2.4	1.0	-	-	-

	Length	7.4	8.4	2.4	1.7	4.3	1.5	-	-	-
	Height	1.8	2.0	1.6	1.5	2.0	1.4	-	-	-
	Depth	7.8	11	16.6	23.5	28	31.4	-	-	-
	Volume	16.0	18.9	1.7	0.9	5.4	0.6	-	-	-
	Q	-	-	-	-	-	-	-	-	-
	G	1	1	-	-	-	-	-	-	-
	M	2	1	-	-	-	-	-	-	-
	W	4	15	6	9	6	2	-	-	-
	P	-	5	-	-	-	-	-	-	-
	L	-	3	-	-	-	-	-	-	-
	Width	5.6	4.2	3.0	4.9	3.5	2.4	-	-	-
9	Length	9.7	6.4	4.2	8.5	6.3	3.8	-	-	-
	Height	2.1	2.0	1.7	1.9	1.5	1.5	-	-	-
	Depth	5.1	7	10.7	13.9	18.5	23.5	-	-	-
	Volume	29.9	14.1	5.6	20.7	8.7	3.6	-	-	-
	Q	-	-	-	-	-	-	-	-	-
	G	-	-	-	-	-	-	-	-	-
	M	-	1	-	-	-	-	-	-	-
	W	5	27	-	11	5	10	-	-	-
	P	-	9	-	-	1	-	-	-	-
	L	-	8	-	-	-	-	-	-	-
	Width	4.8	3.3	4.3	3.8	2.1	3.2	1.8	-	-
10	Length	8.6	4.4	7.5	5.6	2.3	4.1	2.3	-	-
	Height	2.3	2.2	2.0	2.2	1.8	2.0	1.8	-	-
	Depth	6.3	9.2	13	19.2	23.8	29	30.8	-	-
	Volume	24.9	8.4	16.9	12.3	2.3	6.9	2.0	-	-
	Q	-	-	-	-	-	-	-	-	-
	G	-	-	-	-	-	-	1	-	-
	M	2	1	-	1	-	-	-	-	-
	W	9	-	-	64	11	2	11	-	-
	P	12	-	-	6	-	-	-	-	-
	L	5	-	-	2	-	-	-	-	-
	Width	4.7	4.5	3.2	2.6	2.7	3.2	-	-	-
11	Length	9.5	8	4.0	3.5	3.4	5.0	-	-	-
	Height	2.2	2.0	1.8	2.0	1.5	1.9	-	-	-
	Depth	8.2	13	15.8	23	31.5	34.9	-	-	-
	Volume	25.7	18.9	6.0	4.8	3.6	8.0	-	-	-
	Q	-	-	-	-	-	-	-	-	-
	G	-	1	-	1	-	-	-	-	-
	M	-	1	-	-	-	-	-	-	-
	W	12	22	22	-	12	6	-	-	-
	P	-	-	7	-	-	-	-	-	-

	L	-	-	4	-	-	-	-	-	-
12	Width	4.2	4.1	5.2	2.3	2.2	1.6	1.8	1.7	-
	Length	6.4	6.0	9.3	2.5	2.2	1.5	3.4	3.0	-
	Height	2.1	2.0	2.2	1.8	1.5	1.4	1.5	1.4	-
	Depth	7.1	10	13.2	20.8	24.5	29.4	32.5	35.4	-
	Volume	14.8	12.9	27.9	2.7	1.9	0.9	2.4	1.9	-
	Q	-	-	-	-	-	-	-	-	-
	G	-	-	-	-	-	-	1	-	-
	M	4	-	2	-	-	-	-	-	-
	W	1	-	7	3	16	18	7	10	-
	P	-	-	-	18	-	-	-	-	-
	L	-	-	-	14	1	-	-	-	-
13	Width	4.0	3.7	2.2	1.7	3.5	2.5	2.4	1.5	-
	Length	6.8	6.5	2.5	2.0	5.6	3.4	2.4	2.0	-
	Height	2.4	2.1	1.7	1.5	2.0	1.6	1.4	1.3	-
	Depth	6.4	12.1	15.7	18.5	24	26.6	29.4	32.3	-
	Volume	17.1	13.2	2.5	1.3	10.3	3.6	2.1	1.0	-
	Q	-	-	-	-	-	-	-	-	-
	G	-	-	-	-	-	-	-	-	-
	M	-	2	2	-	1	-	-	-	-
	W	-	14	10	4	33	5	1	4	-
	P	-	-	6	-	-	-	-	-	-
	L	-	-	5	-	-	-	-	-	-
14	Width	5.8	4.5	5.4	4.2	3.2	-	-	-	-
	Length	9.3	7.1	10.2	7.6	5.8	-	-	-	-
	Height	2.1	2.1	2.0	1.8	1.8	-	-	-	-
	Depth	7.1	8.1	14	19.8	22.8	-	-	-	-
	Volume	29.7	17.6	28.8	15.0	8.8	-	-	-	-
	Q	-	-	-	-	-	-	-	-	-
	G	1	-	-	-	-	-	-	-	-
	M	1	1	-	-	-	-	-	-	-
	W	21	26	20	4	-	-	-	-	-
	P	10	-	-	-	-	-	-	-	-
	L	8	-	-	-	-	-	-	-	-
15	Width	6.0	5.1	2.2	5.3	4.3	3.9	-	-	-
	Length	12	10.3	3.5	10.3	9.2	7.0	-	-	-
	Height	2.2	2.1	1.7	2.0	2.0	1.9	-	-	-
	Depth	6.2	8.1	11.7	14	22	26.9	-	-	-
	Volume	41.5	28.9	3.4	28.6	20.7	13.6	-	-	-
	Q	-	-	-	-	-	-	-	-	-
	G	-	-	-	-	-	1	-	-	-
	M	3	-	-	-	-	-	-	-	-

	W	-	-	-	8	13	15	-	-	-
	P	1	-	6	-	-	-	-	-	-
	L	-	-	3	-	-	-	-	-	-
	Width	4.7	4.5	3.9	3.4	2.5	2.8	-	-	-
16	Length	7.6	8.4	6.0	4.1	3.3	3.9	-	-	-
	Height	2.1	2.0	1.8	2.0	1.6	1.5	-	-	-
	Depth	6.1	8	11.8	17	19.6	23.5	-	-	-
	Volume	19.6	19.8	11.0	7.3	3.5	4.3	-	-	-
	Q	-	-	-	-	-	-	-	-	-
	G	-	-	1	-	-	-	-	-	-
	M	4	-	3	-	-	-	-	-	-
	W	21	6	24	5	6	13	-	-	-
	P	7	-	21	1	-	-	-	-	-
	L	5	-	15	-	-	-	-	-	-
	Width	5.2	4.1	4.0	3.4	3.0	2.6	2.0	1.5	-
17	Length	9.3	7.5	7.2	5.8	3.4	3.0	2.3	1.8	-
	Height	1.8	1.5	1.8	1.6	1.8	1.5	1.5	1.5	-
	Depth	5.8	7.5	10.8	14.6	17.8	20.5	25.5	29.5	-
	Volume	22.8	12.1	13.6	8.3	4.8	3.1	1.8	1.1	-
	Q	1	-	-	-	-	-	-	-	-
	G	-	-	-	1	-	-	-	-	-
	M	-	-	-	-	-	-	-	-	-
	W	29	22	14	35	19	6	-	-	-
	P	25	-	-	-	-	-	-	-	-
	L	17	-	-	-	-	-	-	-	-
	Width	3.6	4.0	3.7	2.2	-	-	-	-	-
18	Length	5.5	6.3	5.0	3.2	-	-	-	-	-
	Height	2.0	2.0	1.7	1.5	-	-	-	-	-
	Depth	7	9	16.7	20.5	-	-	-	-	-
	Volume	10.4	13.2	8.2	2.8	-	-	-	-	-
	Q	-	-	-	-	-	-	-	-	-
	G	-	-	-	-	-	-	-	-	-
	M	-	-	1	-	-	-	-	-	-
	W	2	8	9	3	-	-	-	-	-
	P	-	1	-	-	-	-	-	-	-
	L	-	-	-	-	-	-	-	-	-
	Width	5.7	4.3	4.0	3.2	2.5	-	-	-	-
19	Length	9.6	6.2	5.7	4.4	3.1	-	-	-	-
	Height	1.9	1.9	1.6	1.5	1.6	-	-	-	-
	Depth	5.9	7.9	12.6	19.5	27.6	-	-	-	-
	Volume	27.2	13.3	9.6	5.5	3.3	-	-	-	-
	Q	-	-	-	-	-	-	-	-	-

	G	-	1	2	-	-	-	-	-	-
	M	-	-	-	-	-	-	-	-	-
	W	10	19	34	4	4	-	-	-	-
	P	1	15	1	-	-	-	-	-	-
	L	3	12	-	-	-	-	-	-	-
	Width	4.6	5.8	4.9	4.3	3.4	3.6	-	-	-
20	Length	7.5	11.8	9.3	8.1	5.5	5.0	-	-	-
	Height	2.2	2.4	2.1	2.0	1.8	1.5	-	-	-
	Depth	8.2	11.4	19.1	23	24.8	28.5	-	-	-
	Volume	19.9	43.0	25.1	18.2	8.8	7.1	-	-	-
	Q	-	-	-	-	-	-	-	-	-
	G	-	-	-	-	-	-	-	-	-
	M	-	2	-	-	-	-	-	-	-
	W	5	3	-	4	6	8	-	-	-
	P	3	1	-	-	3	-	-	-	-
	L	-	-	-	-	-	-	-	-	-
	Width	7.2	6.8	4.6	4.0	3.8	-	-	-	-
21	Length	11.3	11.5	7.4	7.1	5.4	-	-	-	-
	Height	2.3	2.0	1.8	1.7	1.8	-	-	-	-
	Depth	7.3	12	15.8	19.7	23.8	-	-	-	-
	Volume	49.0	41.0	16.0	12.6	9.7	-	-	-	-
	Q	-	-	-	-	-	-	-	-	-
	G	-	-	-	-	-	-	-	-	-
	M	-	5	-	-	1	-	-	-	-
	W	7	3	1	-	3	-	-	-	-
	P	-	-	-	-	-	-	-	-	-
	L	-	-	-	-	-	-	-	-	-
	Width	6.7	3.2	5.0	3.2	4.0	-	-	-	-
22	Length	10.5	4.5	9.0	4.5	6.3	-	-	-	-
	Height	1.7	1.8	2.0	1.6	1.7	-	-	-	-
	Depth	5.7	10.8	13	21.6	28.7	-	-	-	-
	Volume	31.3	6.8	23.6	6.0	11.2	-	-	-	-
	Q	-	-	-	-	-	-	-	-	-
	G	-	-	-	-	-	-	-	-	-
	M	6	-	1	-	-	-	-	-	-
	W	27	3	11	4	2	-	-	-	-
	P	13	-	-	-	-	-	-	-	-
	L	9	-	-	-	-	-	-	-	-
	Width	6.7	6.1	4.1	4.6	4.5	3.7	3.6	-	-
23	Length	12.5	11.2	5.8	7.3	8.5	5.0	5.7	-	-
	Height	2.5	2.2	2.1	2.0	2.0	1.9	1.8	-	-
	Depth	7.5	10.2	14.1	18	22	25.9	29.8	-	-

	Volume	54.8	39.4	13.1	17.6	20.0	9.2	9.7	-	-
	Q	-	-	-	-	-	-	-	-	-
	G	-	1	-	-	-	-	-	-	-
	M	-	1	-	-	-	-	-	-	-
	W	3	3	12	7	-	1	1	-	-
	P	-	-	2	1	-	-	-	-	-
	L	-	-	-	-	-	-	-	-	-
	Width	7.0	6.2	5.7	-	-	-	-	-	-
24	Length	14.1	13.3	10.4	-	-	-	-	-	-
	Height	2.2	2.2	2.1	-	-	-	-	-	-
	Depth	8.2	12.2	23.1	-	-	-	-	-	-
	Volume	56.9	47.5	32.6	-	-	-	-	-	-
	Q	-	-	-	-	-	-	-	-	-
	G	-	-	-	-	-	-	-	-	-
	M	2	-	1	-	-	-	-	-	-
	W	5	-	5	-	-	-	-	-	-
	P	-	-	-	-	-	-	-	-	-
	L	-	-	-	-	-	-	-	-	-
