# Supplementary material for: Nest architecture and colony composition in two populations of Ectatomma ruidum sp. 2 (E. ruidum species complex) in southwestern Colombia
Source: PLoS One. 2022 Feb 2;17(2):e0263382. doi: 10.1371/journal.pone.0263382 (PMC8809609; doi:10.1371/journal.pone.0263382)
Supplement: S3 Table — ‘Cali’: Campus of the Universidad del Valle; ‘Cauca’: Vereda El Rosal, Caldano; n: nest sample size; Q: queens; G: gynes; M: males; W: workers; P: pupae; L: larvae. Student t tests were performed to compare whether the presence or absence of a queen had any effect, with *: p < 0.05; **: p < 0.01; ***: p < 0.001; ****: p < 0.0001. #A Chi-square test was performed to compare the proportion of queenright nests in both localities with χ2 (1, N = 152) = 19.0244, p = 0.000013. (PDF) [file pone.0263382.s003.pdf]

Table S3. Mean (± SEM) nest population and nest depth for all collected nests according to the locality or the presence (queenright) or absence (queenless) of a queen, and at each locality according to the presence or absence of a queen. 'Cali': Campus of the Universidad del Valle; 'Cauca': Vereda El Rosal, Caldano; n: nest sample size; Q: queens; G: gynes; M: males; W: workers; P: pupae; L: larvae. Student *t* tests were performed to compare whether the presence or absence of a queen had any effect, with *: $p < 0.05$; **: $p < 0.01$; ***: $p < 0.001$; ****: $p < 0.0001$. [#]A Chi-square test was performed to compare the proportion of queenright nests in both localities with χ^2 (1, $N = 152$) = 19.0244, p = 0.000013.

	Nest type	n	Q	G	M	W	P	L	Nest depth
All nests	'Cali'	103	12/103	0.9 ± 0.1	1.8 ± 0.2	65.4 ± 3.3	11.5 ± 1.0	25.6 ± 2.1	28.7 ± 0.5
	p		**** [#]	NS	NS	****	****	****	***
	'Cauca'	49	21/49	0.1 ± 0.0	1.1 ± 0.4	99.1 ± 7.6	57.0 ± 7.0	74.2 ± 6.8	35.4 ± 1.8
	Queenright	33	1	0.2 ± 0.1	1.0 ± 0.5	135.1 ± 7.9	57.9 ± 9.3	86.8 ± 9.0	37.1 ± 2.3
	p			***	NS	***	***	***	***
	Queenless	119	0	0.7 ± 0.1	1.7 ± 0.2	60.0 ± 2.3	17.4 ± 2.1	28.7 ± 2.1	29.1 ± 0.5
'Cali'	Queenright	12	1	0.4 ± 0.1	0.2 ± 0.2	129.3 ± 11.6	11.3 ± 2.7	49.8 ± 9.8	30.5 ± 1.7
	p			*	*	***	NS	***	NS
	Queenless	91	0	0.9 ± 0.2	2.0 ± 0.2	57.0 ± 2.3	11.5 ± 1.1	22.4 ± 1.7	28.5 ± 0.5
'Cauca'	Queenright	21	1	0.0 ± 0.0	1.5 ± 0.7	138.5 ± 10.6	84.5 ± 10.8	107.9 ± 10.7	40.9 ± 3.2
	p			NS	NS	**	**	**	**
	Queenless	28	0	0.1 ± 0.1	0.8 ± 0.5	69.6 ± 6.4	36.3 ± 7.1	49.0 ± 5.1	31.3 ± 1.7
