# Supplementary material for: Nest architecture and colony composition in two populations of Ectatomma ruidum sp. 2 (E. ruidum species complex) in southwestern Colombia
Source: PLoS One. 2022 Feb 2;17(2):e0263382. doi: 10.1371/journal.pone.0263382 (PMC8809609; doi:10.1371/journal.pone.0263382)

**Figure S1. Relationship between different categories of nest population for the 152 complete nests of *E. ruidum* sp. 2. extracted.** (A) between the total number of workers per nest and the total number of larvae ($R_{(152)} = 0.6976$, $p < 0.001$) or (B) the total number of pupae ($R_{(152)} = 0.6050$, $p < 0.001$) and (C) between both categories of brood ($R_{(152)} = 0.7252$, $p < 0.001$).

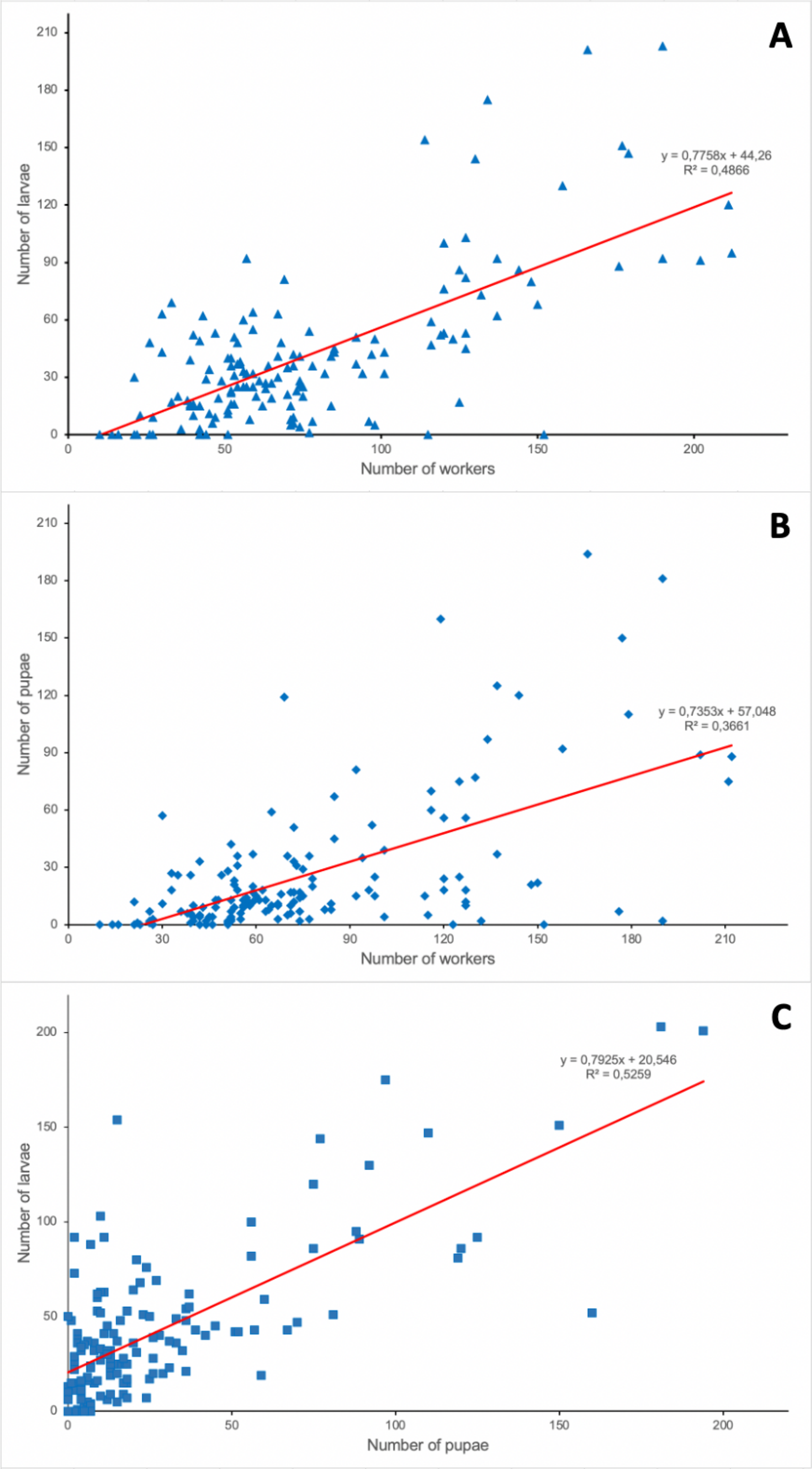

Supplement: S1 Fig — (A) between the total number of workers per nest and the total number of larvae (R(152) = 0.6976, p < 0.001) or (B) the total number of pupae (R(152) = 0.6050, p < 0.001) and (C) between both categories of brood (R(152) = 0.7252, p < 0.001). (PDF) [file pone.0263382.s004.pdf]
