# Supplementary material for: Nest architecture and colony composition in two populations of Ectatomma ruidum sp. 2 (E. ruidum species complex) in southwestern Colombia
Source: PLoS One. 2022 Feb 2;17(2):e0263382. doi: 10.1371/journal.pone.0263382 (PMC8809609; doi:10.1371/journal.pone.0263382)
Supplement: Attachment — Submitted filename: Response to Reviewers(PONE-D-21-13547R1).pdf [file pone.0263382.s007.pdf]

# Response to reviewers

PONE-D-21-13547R1
Nest architecture and colony composition in two populations of Ectatomma ruidum sp. 2 (E. ruidum species complex) in southwestern Colombia
PLOS ONE

Dear Dr. Lachaud,

Thank you for submitting your manuscript to PLOS ONE. After careful consideration, we have decided that your manuscript does not meet our criteria for publication and must therefore be rejected.

I forwarded the manuscript to the reviewers again and received input from two reviewers.
After consideration of all revisions, I have decided that your manuscript does not reach scientific soundness for publications.

I am sorry that we cannot be more positive on this occasion, but hope that you appreciate the reasons for this decision.

Yours sincerely,

Maykon Passos Cristiano, D. Sc.
Academic Editor
PLOS ONE

---

Reviewers' comments:

Reviewer's Responses to Questions
**Comments to the Author**

1. If the authors have adequately addressed your comments raised in a previous round of review and you feel that this manuscript is now acceptable for publication, you may indicate that here to bypass the "Comments to the Author" section, enter your conflict of interest statement in the "Confidential to Editor" section, and submit your "Accept" recommendation.
Reviewer #3: All comments have been addressed
Reviewer #4: All comments have been addressed

---

2. Is the manuscript technically sound, and do the data support the conclusions?

The manuscript must describe a technically sound piece of scientific research with data that supports the conclusions. Experiments must have been conducted rigorously, with appropriate controls, replication, and sample sizes. The conclusions must be drawn appropriately based on the data presented.
Reviewer #3 : Yes
Reviewer #4 : Yes

---

3. Has the statistical analysis been performed appropriately and rigorously?
Reviewer #3 : Yes

Reviewer #4 : Yes

---

4. Have the authors made all data underlying the findings in their manuscript fully available?

The PLOS Data policy requires authors to make all data underlying the findings described in their manuscript fully available without restriction, with rare exception (please refer to the Data Availability Statement in the manuscript PDF file). The data should be provided as part of the manuscript or its supporting information, or deposited to a public repository. For example, in addition to summary statistics, the data points behind means, medians and variance measures should be available. If there are restrictions on publicly sharing data—e.g. participant privacy or use of data from a third party—those must be specified.
Reviewer #3 : Yes
Reviewer #4 : Yes

---

5. Is the manuscript presented in an intelligible fashion and written in standard English?

PLOS ONE does not copyedit accepted manuscripts, so the language in submitted articles must be clear, correct, and unambiguous. Any typographical or grammatical errors should be corrected at revision, so please note any specific errors here.
Reviewer #3 : Yes
Reviewer #4 : Yes

---

6. Review Comments to the Author

Please use the space provided to explain your answers to the questions above. You may also include additional comments for the author, including concerns about dual publication, research ethics, or publication ethics. (Please upload your review as an attachment if it exceeds 20,000 characters)

Reviewer #3: Dear authors,
The manuscript was improved and the majority of questions and suggestions has been explained or corrected in the main text. Now the mansucript can be accepted for publication.
R: We thank the reviewer. The comment is sufficiently explicit and clear and does not require any specific response from us.

Reviewer #4: The authors have greatly improved this paper since the first review. I understand that they did not always have the data to follow some of my suggestions, but they did a good job in bringing the message across. I have very few suggestions for improvement.

I had to look up the meaning of gangue. I don't think I would have used the word in that context, but it is colorful. I doubt it is often used outside the context of mining, but then, you never know how many miners will read this paper.
R: We particularly appreciated the gently mocking tone of this comment. Not being English speakers ourselves, we followed the advice of the English colleague who revised our text, but we have to admit that the term "gangue" is perhaps too specific to mining, even if this term is perfectly appropriate in our respective languages of origin (Spanish and French). In our revision, we have replaced "earthy gangue " with "soil matrix " which is perhaps more commonly used and understandable to English speakers (see lines 142 and 157 in the new revised version).

Line 265. This calculates to 4.6 nests per queen. Couldn't you claim that the average polydomous colony has 4.6 nests? Or do you think some colonies are truly queenless? You even state that there are no gamergates or ergatoid queens, so doesn't it follow that there are 4.6 nests per polydomous colony? Obviously, it would be nice to know the range, but at least you have a mean.
R: It is true that, based on our data, we can propose that an average polydomous colony has 4.6 nests even though the precise range is difficult to specify. However, as indicated in manuscript PONE-D-21-13547R1 (see lines 432-435 of the previous version), at least some of these queenless nests truly reflect a natural phenomenon of queen death or colony weakening. We have added a few lines in the discussion to take this comment into account (see lines 441-448 in the new revised version).

Line 346 and 388. Why not make an estimate of the amount of soil excavated by your population, especially since you did a second count and discovered many new nests? The mean weight and depth of the bioturbation rate could be estimated.
R: The reviewer is absolutely right. Therefore, we have added in the discussion the requested bioturbation estimate (in kg of dry soil per hectare per day) taking into account the available values on our population (see lines 346-351 of the new revised version).

There are too many references. I suggest culling the list to be more targeted and relevant to the subject. This is not a review article.
R: We have done our best to address this comment by reducing the total number of citations by over 20%.

---

7. PLOS authors have the option to publish the peer review history of their article (what does this mean?). If published, this will include your full peer review and any attached files.

If you choose “no”, your identity will remain anonymous but your review may still be made public.

**Do you want your identity to be public for this peer review?** For information about this choice, including consent withdrawal, please see our Privacy Policy.

Reviewer #3: No
Reviewer #4: No
