# Supplementary material for: Nest architecture and colony composition in two populations of Ectatomma ruidum sp. 2 (E. ruidum species complex) in southwestern Colombia
Source: PLoS One. 2022 Feb 2;17(2):e0263382. doi: 10.1371/journal.pone.0263382 (PMC8809609; doi:10.1371/journal.pone.0263382)
Supplement: Attachment — Submitted filename: ReviewPlosOne_JLopes.docx [file pone.0263382.s005.docx]

**General comment**

The paper brings information about the nest architecture of an ant species of economic importance in Colombia, which are relevant to this research field. However, the writing is very confusing and the applied analyses and their interpretation are very poor. It is necessary to reanalyze and rewrite the text. The discussion must be more reliant on your own results, instead of only existing literature.

**Abstract**

This section lacks an introduction with the theorical background that justifies your study. Also, you say in this section that you have strong correlations between the variables, but any correlation is near 90%. The conclusion here is much better than what is presented at the end of the discussion, but you need to write and present your data and analyses in a way that you can make the readers agree with you.

**Introduction**

The text of this section is too long and takes too long to approach the issue of the study. For example, the first paragraph has 27 lines and addresses different points concerning the nest architecture, such as how the construction material interferes with internal temperature, strategies to avoid flooding and also thermal preferences of individuals inside the nest. These issues are presented without any cohesion, making the text awkward. Also, the English needs improvement.

The second and third paragraph seem like a review and up to there I can not know what is the point of your study. And even with all these information, the importance/justificative of knowing the relation between nest architecture and population for these ant species is not presented.

Figure 1A is really very beautiful, but it does not illustrate the “high bioturbation impact during nest excavation” as is called in the text. The same is true for the figure caption “nest excavation”. What I see is an ant transporting a pellet of soil (?) with its mandibles. The same goes for the other photos of Figure 1. What do you want to show with these photos? The figure 1 is once again called for the images B, C and D at material and methods (line 151), but I still cannot see why the feeding habits are relevant to your study.

The most important justificative of your study is presented only after line 110, which is related to question: Are the nests monodomous or polidomous? On my point of view this is the main justificative of your study. I recommend you to shorten the text before this point and highlight this issue.

In the last paragraph, you first presented two objectives:

- to evaluate the spatio-temporal competitive relationships of their nests
- to validate, or not, the existence of a possible polydomy.

Following, you presented as objective what actually is your methodology. And then you presented some questions. The first one I can not understand at all. Please clarify. The others seem unrelated with your proposed objectives (listed above). Please clarify how answering these questions can help you reach your objective.

**Material and Methods**

Line 163-164. Why did you sample the nests in 2 different seasons? Furthermore, why did you choose these seasons? Please, justify.

Line 165. How did you mark the nests?

Line 166-170. You report that from 188 nests, 15 were not used as data due their small population. Following, you state that you molded 24 nests. What happened to the others? Please explain.

Line 179. Change “nests” for “chambers”.

Line 188-189. Why were the nests from Vereda El Rosal not molded?

Line 193. What exactly did you mean with chamber size? Chamber area? Chamber volume? Size is too vague. Please be more specific about what kind of data you analyzed.

Line 193-194. “Student-*t* tests were performed to compare whether the presence or absence of queens had any effect …” Effect of what over what?

Also, in the section Data Analysis you must tell the readers which was your random variable in the GLMM, which program and packages of statistical analyses you used. There are a lot of information that are lacking in this section.

**Results**

In general, the applied statistical analyses and specially their interpretations are not adequate (see detailed comments below). I strongly suggest that instead of doing a lot of paired correlations, you consider applying a multivariate analysis, in which you can verify which variables were associated to the similarities and dissimilarities between the nests. Even the locale can be incorporated in the multivariate analysis, making your result more robust. This way you can examine several variables (population parameters) to see if one or more of them are predictive of a certain outcome, in this case the nest architecture.

Line 242. “The tunnels walls and the floors of the chambers were lined with a very compact and thin surface …” This is very confusing to read and understand, please review the English writing.

Line 245-246. The negative correlation between chamber volume, not size, and depth does not mean that superficial chambers are larger. Instead, the analysis indicates that as the chamber was deeper, its volume was smaller. Also, the R value (-0.62) is too small. Please interpret the analysis according to the results it gives to you.

Lines 246-250. What is the MLGM analysis? It was not described in material and methods. Also, which chambers did you consider as the upper ones? The third chamber is not one of these upper chambers. Once again you can not say that the 3^rd^ chamber was smaller than the 2^nd^ or the 1^st^, as you did not apply any statistical test.

At Table 2, what does “nb” mean after the word worker or larvae? Here there is another statistical problem. Queen presence and population should not be analyzed with a linear regression, but instead with a logistic one, as presence/absence of the queen is a binomial variable.

**Discussion**

Just like the introduction section, the text is too long. The ideas are not well coordinated. Also, I can not find the answers to the proposed questions addressed at the end of the introduction. Especially in relation to the status of the species’ polidomy or monodomy. You suggested at the last line that they were probably monodomous, based in the fact that there are queenless nests. However, you did not find horizontal connecting tunnels among nests or any evidence of external communication. Thus, in which evidences is your answer based on? I recommend that while restructuring this section, you follow your proposed questions.
